# “Like Nothing I’ve Seen Before”: A Qualitative Inquiry Into the Lived Experience of Competing in a Trail Running Event

**DOI:** 10.3389/fpsyg.2022.817685

**Published:** 2022-03-14

**Authors:** Timothy P. Chambers, Jennifer Poidomani

**Affiliations:** ^1^School of Psychology, Deakin University, Geelong, VIC, Australia; ^2^Discipline of Psychological Science, Australian College of Applied Psychology (ACAP), Sydney, NSW, Australia

**Keywords:** green exercise, interpretative phenomenological analysis, ecological dynamics, nature, connection

## Abstract

**Background:**

A recent upsurge in nature-based exercise research demonstrates the potential added benefits of exercising in this context compared to more urban ones. Yet there is a lack of qualitative research investigating the lived experiences of those who participate in nature-based exercise events.

**Objective:**

To explore the lived experience of individuals who were first-time participants in a nature-based running event.

**Method:**

Six participants who completed the Run Forrest trail run for the first time were individually interviewed. Semi-structured interviews were devised, and participants were invited to talk about their experiences of running in the event. Interviews were analysed using interpretative phenomenological analysis.

**Results:**

Following data analysis, two superordinate themes were constructed to resemble participant experiences. “Maintaining good health is vital” reflected participants’ personal theory of health and their perceived benefits of exercise. “Nature as a special place” reflected the atmospheric characteristics of the event, including the pre-event set-up and the actual running event itself.

**Conclusion:**

Analysis suggests that participants considered the event and natural environment to provide unique value adding opportunities that encouraged positive experiences. These results also reaffirm the positive benefits associated with nature-based exercise, including potential benefits to individual wellbeing. Further research in this context may strengthen our collective understanding regarding individual motivation towards such events.

## Introduction

Physical inactivity is the fourth leading risk factor for global mortality (WHO, 2014). Associated health problems such as cardiovascular disease, diabetes, obesity, and mood disorders not only affect an individual’s quality of life but also create a substantial economic burden (Ding et al., 2016). The implications of inactivity are therefore cause for concern, prompting reviews of public policies and initiatives to combat the risks associated with inactive lifestyles (WHO, 2014). Much research has been dedicated to exploring the physical and psychological benefits of physical activity and exercise (Warburton et al., 2006; Reiner et al., 2013), including examining the types of activity undertaken (e.g., Sullivan et al., 2012), and investigating outcomes for clinical (Josefsson et al., 2013; Stanton and Reaburn, 2013) and non-clinical groups (Chang et al., 2012). In the last decade, an area of exercise psychology research that has proliferated is the role of the natural environment.

As humans move rapidly towards an ever-increasing urbanised way of life, researchers across various domains are championing the need for us to reconnect with nature (Frumkin et al., 2016; Bratman et al., 2019). A wealth of evidence already suggests that even brief periods of exposure to nature can benefit individuals with physical (e.g., high blood pressure) and mental (e.g., depression) health issues (Shanahan et al., 2016), with these findings also informing policy change (Pretty et al., 2007). It follows then that nature-based exercise (i.e., any purposeful and planned physical activity that takes place in nature), can lead to better physical and mental health outcomes (Fraser et al., 2019). At times, these benefits can be greater than those reported in other exercise environments, such as the gym or an urban setting (Coon et al., 2011; Yeh et al., 2017; Lahart et al., 2019). Despite the wealth of evidence in favour of being exposed to, and exercising in, the natural environment, there is an emerging critical issue associated with this line of inquiry. Specifically, some researchers are contesting the romanticism associated with nature-based research (van den Berg et al., 2007; Olive and Wheaton, 2021). Rather than further validating the outcomes associated with time in nature, more critical explorations of who accesses what natural environments for what reasons are required. Therefore, investigating the various contexts associated with nature-based activities remains a prominent line of inquiry. One such context that remains unexplored, is natured-based mass participation sporting events. Whilst urban equivalent events (Bauman et al., 2009), which focus on participation and engagement for non-elite participants and can build greater autonomous motivation following the event (Coleman and Sebire, 2016), are increasingly popular (Buning and Walker, 2016), little is known about nature-based mass participation sporting events. In this study, we therefore explored participants’ experiences of competing in a mass-participation trail running event.

Existing natured-based exercise literature is typically underpinned by one of three prominent theories: attention restoration theory (ART; Kaplan and Kaplan, 1989), stress reduction theory (SRT; Ulrich, 1983), and the biophilia hypothesis (Wilson, 1984). All three theories share a similar theoretical stance; nature is seen as an external entity that provides the necessary favourable stimuli that can result in physical and psychological benefits. ART is based on the notion that people have a limited cognitive processing capacity (e.g., attention) that experiences fatigue when in an urban environment (Kaplan and Kaplan, 1989). Exposure to the novel stimuli found in nature can restore attention capabilities (see Kaplan and Kaplan, 1989). SRT builds on ART by suggesting that, in addition to cognitive processes, biological and evolutionary elements are activated when taking in natural environmental features (Ulrich, 1983). The environmental features are thought to lead to a decrease in physiological arousal and restoration of attention (see Ulrich, 1983). The biophilia hypothesis is grounded in an evolutionary perspective whereby humans are considered to have a universal and innate affection towards nature, guiding a tendency to seek it out and benefit from it (Grinde and Patil, 2009). Together, the theories have aided researchers to assess the influence of experimentally manipulated environments on individual performance and wellbeing (Akers et al., 2012; Berman et al., 2012; Araujo et al., 2019). For example, laboratory-based examinations of attention (Roe and Aspinall, 2011; Lee et al., 2015; Neidemeier et al., 2017), task performance (Berman et al., 2008; Rogerson et al., 2016), and physiological biomarkers (Kjellgren and Buhrkall, 2010; Neidemeier et al., 2017; Olafsdottir et al., 2017) have yielded results that support the relative theories. Collectively, the findings suggested that exposure to nature (i.e., simulated or real) improved performance and subjective wellbeing above exposure to urban environments (see Coon et al., 2011 for analysis).

Despite yielding promising findings, criticisms of the nature-based exercise research are vast. For example, an emphasis on quantitative laboratory-based experiments, small sample sizes, overrepresentation of university students, unclear procedures and identification of green environments, insufficient statistical power, and a lack of longitudinal research, have all been offered as factors that make the interpretation of findings difficult (e.g., Lahart et al., 2019). Similarly, the biological deterministic underpinning of the existing theories has been criticised as too simplistic and unclear when accounting for complex human behaviour (Joye and de Block, 2011). As such, it is argued that reducing behaviour to a set of predetermined variables that solely promote genetic fitness, fails to account for the subjective and psychological elements that may contribute to nature-based exercise experiences (Martyn and Brymer, 2014; Lawton et al., 2017; Araujo et al., 2019). These limitations result in a failure to account for any identification as to how internal processes (i.e., intention and self-determination) are derived from external processes (i.e., the environment; Araujo et al., 2019), and a general lack of understanding as to how individuals interact holistically with the natural environment. To address these limitations, a contemporary theory has been put forth to encourage researchers to develop a deeper understanding into the nature-based exercise experience.

The ecological dynamics perspective (EDP; Yeh et al., 2016) is grounded in both ecological psychology and dynamics systems theory, suggesting that behaviour is both motivated and shaped by a complex interaction between the individual, task, and the environment. Central to EDP is Gibson’s (1979) notion of affordances, defined as “invitations for behaviour that exist in an environment and depend on the individual’s capacity for action” (Yeh et al., 2016, p 950). According to EDP theorists (e.g., Araujo et al., 2019), affordances provide individuals opportunities to interact physically, psychologically, and emotionally with the environment. Furthermore, the interaction between affordances, active participation, and the individual (i.e., their knowledge, past experiences, thoughts, and capabilities) combine to provide the nature-based exercise experience. Whether this experience results in the reported benefits will depend greatly on the aforementioned factors systematically intertwining at different occasions. The experiences therefore will be subjective and different for all, leading researchers to acknowledge that different measures may be needed when investigating the benefits of nature-based exercise.

There are limited studies to date using an EDP lens in nature-based exercise research, however, the available investigations have shed some light into the experiences of nature-based exercise. For example, qualitative and mixed method approaches have found that participants who demonstrated a high nature-relatedness were more inclined to engage in outdoor physical activity, had lower levels of anxiety, and increased wellbeing (Lawton et al., 2017). Similarly, a connection to nature was associated with lower levels of state and trait anxiety (Martyn and Brymer, 2014). To tease out the understanding of the human-nature relationship further, Schweitzer et al. (2018) explored participants lived experiences of the natural world and noted that the human-nature relationship can be conceived using psychoanalytic concepts. These authors suggest that nature not only provides cognitive and emotional restorative benefits, but it is also an integral part for the participant’s sense of self (Schweitzer et al., 2018). These findings suggest that people connect and relate with nature at a deep psychological and emotional level, drawing significant personal meaning from nature interactions which act as contributing factors mediating whether people participate in outdoor physical activity and experience the benefits of nature-based exercise (Martyn and Brymer, 2014; Lawton et al., 2017; Schweitzer et al., 2018).

Other EDP researchers have focussed primarily on applying the conceptual framework to the understanding of nature-based sporting events in relation to participant wellbeing (Immonen et al., 2018; MacKenzie and Brymer, 2018). For example, MacKenzie and Brymer (2018) suggest adventurous nature sports events provide participants opportunities for participant-environment challenge, skill development, social interaction, and connections to the natural world which are important psychological needs. Theoretically, these authors suggest that sporting events hold an important place in facilitating the opportunity to fulfil these needs. While there is evidence outside of the nature-based exercise context to suggest event attributes (i.e., opportunities for accessibility and inclusivity, personal gain and helping others; Stevinson et al., 2015) are factors that encourage health-enhancing behaviours leading to positive wellbeing, further research is needed to assess nature-based event opportunities and how they may impact or facilitate participant wellbeing.

### The Present Study

Physical inactivity remains a global concern. The benefits of being physically active are extensive (Warburton et al., 2006), including exercising in nature (Pretty et al., 2005), and efforts to increase activity at an individual and societal level abound. Trail running events, which fall under the category of mass-participation sporting events, are one such initiative. Whilst there is a growing body of literature on the myriad of individual (e.g., improved mental health; Dunne et al., 2021; Quirk et al., 2021) and social (e.g., increased social connections; Sharman et al., 2019; Davis et al., 2021) benefits associated with *parkrun*, to date little research has investigated participants’ lived experiences of completing a trail running event. Given the known benefits associated with nature-based exercise, and the increasing popularity of such events, the purpose of this study was to explore the lived experiences of individuals who completed their first trail running event. The current study was supported by an EDP to better understand the human-nature relationship in this context, with three research questions guiding the study: (i) what are the lived experiences of people who participate in a nature-based trail running event? (ii) how do people engage with nature during this kind of event?, and (iii) what motivates individuals to take part in this kind of event?

## Methodology

### Design

When conceptualizing the current study with the aim to understand and explore the lived experiences of running in nature, we maintained an interpretivist view that enabled us to explore and understand the unique and subjective experiences of individuals (Scotland, 2012). Accordingly, we acknowledged that there is no one single correct route to, or employable method to, acquire knowledge. In light of this, the researchers considered the methods which would yield the most interesting and relevant outcomes for the field of inquiry (Cacioppo et al., 2004; Thomas and Hodges, 2010; Scotland, 2012). To determine this, we turned to contemporary qualitative research in sport and exercise as a guide. Given that several recent investigations in this domain also adhered to an interpretivist paradigm (Olafsdottir et al., 2017; Sandardos and Chambers, 2019), we believed that this approach was the most appropriate for our area of inquiry.

From an interpretivist epistemological stance, it is understood that knowledge and ways of knowing are a result of the interactions between human consciousness and participation with the world and others (Grix, 2004; Scotland, 2012). There is no one universal truth to knowledge; it is simultaneously moulded and shaped by those who are experiencing and constructing it (Scotland, 2012). Within research, the inquirer and the inquired-into are interlocked in an interactive process whereby knowledge and meaning are co-constructed through talking and listening (Mustafa, 2011).

Our ontological position is relativism (Scotland, 2012). From this stance it is thought that there are multiple, subjective realities which are mediated by our senses (Guba and Lincoln, 1994). Our experiences of reality are actively shaped and transmitted through our use of language and interactions with others (Crotty, 1998). These realities can be explored through an interaction between the researcher and the participant in meaningful ways to discover how people make sense of and experience their realities (Crotty, 1998).

#### Philosophical Base and Rationale for Chosen Method

The philosophical base underlying the methodology of interpretive research involves the use of hermeneutics, phenomenology, and an idiographic focus (Boland, 1985; Scotland, 2012). Interpretive methodology also includes the use of broad research questions, whereby knowledge is generated *via* the accumulated data and not preceding it (Cohen et al., 2007). Therefore, researchers must make their agenda and beliefs apparent before beginning research and during the interpretation of the data (Edge and Richards, 1998). The interpretivist methodology also upholds that events are not reduced to simplistic interpretations. Rather, new layers of understanding are sought, and it is the researcher and participant interaction that is the key to gaining this understanding (Creswell, 2009; Scotland, 2012).

Hermeneutics is both an approach to understanding humans as well as a mode of analysis in research (Gadamer, 1976). The essential principle of hermeneutics is that all understanding is achieved by considering both the individual meanings of parts as well as the whole that is formed (Gadamer, 1976). Adopting hermeneutics enables researchers to understand the social context by which human beings navigate, construct and experience the world as well as, identify the hidden meanings derived in the language used to express experience (Creswell, 2009). Phenomenology is intended to discover and express parts of phenomena as they appear to be (Creswell, 1998). Described as the study of the lived experiences of people regarding a concept or phenomenon (Creswell, 1998), phenomenology research refers to the gathering of deep, rich, and contextual data that can represent the perceptions of the research participant (Lester, 1999). An idiographic focus in interpretivist research describes the aim to investigate a phenomena from the unique perspective of an individual (Scotland, 2012). One method that aligns well with the interpretive paradigm and has been used in contemporary sports, exercise and natural environment literature is interpretative phenomenological analysis (IPA; Smith et al., 2009; Stansen and Chambers, 2017; Sandardos and Chambers, 2019).

Interpretative phenomenological analysis uses an inductive approach by which the inquirer and inquired-into enter a hermeneutic circle (Smith et al., 2009). This interaction is an integral process by which the inquired-into is seen as the expert of their knowledge and experiences and it is the role of the inquirer to gain an understanding of their insight (Smith et al., 2009). The hermeneutic circle is intended to draw out the idiographic descriptions of an event or phenomenon (Eatough and Smith, 2008; Smith et al., 2009; Smith, 2019). By drawing out rich and contextual information, the inquirer and inquired-into can explore the subjective meaning assigned to the experience from the unique perspective of the person who experienced it (Smith et al., 2009). In other words, IPA lends well to the investigation of people’s perceptions of a significant experience (Smith, 2019). Accordingly, it is common for IPA studies recruit small sample sizes (e.g., 4–6 participants; Smith, 2017) that enable the idiographic descriptions of participant experiences. Given the alignment of IPA with the interpretivist paradigm and previous applications of IPA in current sports, exercise, and natural environment literature (e.g., Stansen and Chambers, 2017; Sandardos and Chambers, 2019), and the intention to explore a unique personal experience, we perceived this method to be the most appropriate for the current study.

### The Event

To investigate why and how participants engaged with a nature-based running event, we chose the Otway Ranges Run Forrest event. The event was based in Forrest, which is located in the Otway national park in regional Victoria, Australia, and is approximately a 2.5 h drive from Melbourne. Commencing in the Forrest township, affectionately termed the gateway to the Otways (Visit Victoria, 2020), the trail run heads south towards the west Barwon reservoir, then east towards Lake Elizabeth, before returning to Forrest. Participants can complete either a 10 or 21 km run. The course is labelled as “tough and tight” as participants traverse along a mixture of fire breaks, mountain bike tracks, nature walking trails, and foot bridges all the while being immersed in the natural surrounds (Southern Exposure, 2020).

### Participants

Six participants took part in the present study. Participants were recruited using a purposive sampling technique (Patton, 2002). Inclusion criteria required that all participants were over the age of 18, were regular exercisers (e.g., 150 min of exercise per week), first-time participants of the Otway Run Forrest event, and provided written consent. Participation was voluntary. Table 1 represents participant demographic information, including the pseudonyms, gender, age ranges, and distance run of the six participants. In order to protect participants’ identities, no further demographic information was collected.

**TABLE 1:** Participant demographic information.

	Participant demographic information		
Pseudonym	Gender	Age-range (yrs.)	Distance run (km)
Sally	Female	50–60	10
Jane	Female	70+	10
Rachel	Female	30–40	10
Susan	Female	30–40	21
Molly	Female	40–50	10
Mike	Male	50–60	10

### Interview Protocol

Based on recommendations from previous IPA researchers, a semi-structured interview schedule was devised to guide the interviews (Smith et al., 2009). Questions were drawn from the available relevant literature and the researchers’ personal experience. Interview questions were open-ended to draw out rich, descriptive, and detailed information. The schedule commenced with rapport building questions (e.g., can you tell me a little bit about your interest in exercise?), before moving into questions designed to explore the details of the lived experiences prior to (e.g., can you describe to me what motivated you to enter this event?), during (e.g., can you walk me through your run from start to finish?), and following the event (e.g., how did you feel after completing the event?). Probes were used in some interviews to encourage further exploration of responses (e.g., can you tell more about the difficult parts of the race?). All interviews were digitally recorded.

Participant interviews were scheduled during August 2019, approximately 6 weeks following the trail running event. The six semi-structured interviews were conducted at a mutually agreed upon time; four were conducted using Zoom (i.e., an online collaborating tool), and two were conducted *via* telephone. One online interview was split over two separate occasions at the participant’s request. All interviews were conducted with a commitment to the idiographic focus and inductive approach emphasized by the interpretive phenomenological analysis method. As such, interviews were one-on-one and in-depth to allow the participant to talk freely and be heard (Smith et al., 2009). The interviews lasted from 30 to 45 min in duration.

### Procedure

Ethical approval was obtained from JP’s institution (#477240419) before researchers contacted the event organisers. The authors received approval to share a research advertisement outlining the current study on the event Facebook page. Interested participants contacted the researchers *via* email. Participants who met the inclusion criteria were sent an information pack (e.g., plain language statement, interview questions, consent form, withdrawal form, and contact details of service providers), and asked to return a consent form. Following written consent, a mutually agreeable time was selected for an interview. All participants were provided a second copy of the interview questions prior to interviewing to familiarize themselves with the questions (Smith et al., 2009). Before interviewing, participants were reminded that participation was voluntary, that they could withdraw at any time, and that the interview would be recorded for transcription. Following the interview, participants were debriefed, informed of the subsequent steps of the researcher (e.g., further contact for member reflection, Smith et al., 2009), and thanked for their time. Interview recordings were sent to an external company for transcription immediately following the interviews. Audio recordings were deleted after the researchers checked the transcription for accuracy.

### Analytical Strategy

Our IPA analysis was guided by a series of steps as outlined in the related methodological literature (e.g., Braun and Clarke, 2013; Sparkes and Smith, 2014). First, the interviews were transcribed as previously mentioned. Second, TC and JP read a transcript (i.e., the first case) many times for familiarization. Third, TC and JP made initial notes of literal, descriptive, and conceptual features of participants’ stories (Braun and Clarke, 2013). As there are no set rules regarding what is to be noted (Sparkes and Smith, 2014), our noting was informed by our personal judgements. Fourth, guided by the initial noting and researcher discussions, TC and JP constructed a series of themes that represented the various patterns of shared meaning we identified in the participants’ stories (Braun and Clarke, 2013). Fifth, TC and JP explored similarities within each narrative and, after a higher level of abstraction, connected the previously constructed themes into superordinate themes. To do so, we created a table of the themes that enabled us to visually connect related themes. Sixth, JP repeated the previous steps for the remaining transcripts. Once we completed these steps, TC and JP explored the case-specific themes across the entire data set to look for other possible interpretations, and subsequently developed a representative map of the findings (Smith and Osborn, 2008; Braun and Clarke, 2013).

### Trustworthiness

As researchers we were committed to establishing rigor that honours contemporary qualitative research practices when using a relativist position (Sparkes and Smith, 2009; Smith and McGannon, 2018). We make clear that the trustworthy techniques used are not intended to demonstrate evidence that our interpretations and analyses are correct according to a set standard for achieving reliability and validity. As we stand, there can be no theory-free knowledge or one correct interpretation of reality. We instead endeavour to illustrate transparency and demonstrate that we have explored as many possible interpretations as time allowed (Smith and McGannon, 2018). On this point, we choose to provide greater transparency regarding our professional backgrounds to help readers understand our position regarding the analysis and interpretation of results. TC is a sport and exercise psychology academic with a background in high-performance sport. As an avid runner (at times on trails), TC has a strong interest in understanding individual’s experiences of exercise in nature. JP is a post-graduate student studying to become a registered psychologist. Whilst JP is not a runner herself, she has an affinity with nature and a family connection to trail running. Building upon this research transparency, we also employed strategies most relevant to our position: a critical friend, researcher reflexivity, member reflection, and crystallization.

#### Critical Friend

TC, who did not partake in interviewing, acted as the critical friend to encourage exploration of different interpretations. In this role, TC was able to provide dispassionate oversight and dialogue surrounding interpretation of the data (Smith and McGannon, 2018). Dialogue between the authors also facilitated researcher reflexivity; these discussions served as a prompt for JP to reflect upon her implicit knowledge, assumptions, and biases throughout data collection and analysis. This approach was taken to better support JP who, as a research student, possessed limited experience in qualitative analysis (Cowan and Taylor, 2016).

#### Reflexivity

JP used TC as a critical friend, an impartial support group, crystallization, and journal to monitor and explore her reactions, judgements, and reasoning during the study (Badbury-Jones, 2007; Berger, 2015). During all stages of the research, JP discussed and explored her personal views and assumptions of all aspects of the study (e.g., views on the research question, the interviewing experience, the participants, the interpretation of the data, and the findings) with herself and others. This technique was used to bring awareness of JP’s situatedness (e.g., age, gender, experiences with exercise in nature, emotional responses, and cultural and philosophical views), and how these features may impact and shape the attainment of knowledge (Berger, 2015). TC encouraged JP to employ reflexivity as an opportunity for JP to learn and develop her research skills.

#### Member Reflections

Member reflection was adopted as a trustworthiness technique that would assist the researchers in gathering a deeper and richer understanding of the participant’s lived experiences (Smith and McGannon, 2018). Participants were sent a copy of their transcripts and analysis of the emerging themes, and were invited to elaborate, correct, or delete any of the information. This technique allowed for transcript accuracy, opportunity to explore and acknowledge differences in interpretations, and facilitate inclusion and transparency in research (Smith and McGannon, 2018). This technique also allowed the researcher the ability to maintain rapport and demonstrate dignity and respect towards participants (Smith and McGannon, 2018). Three of the six participants responded back to the researcher and no further data was obtained.

#### Crystallization

To embrace the many truths indicative of an interpretivist approach, the researchers adopted crystallization as a trustworthiness technique (Richardson, 2000). This technique allowed researchers the opportunity to see many views by applying intuition, creativity, and immersion with the data at all stages (Stewart et al., 2017). In practice, we reflected on thoughts and considerations of the research question, philosophical underpinnings, analysis, and findings both together and separately to achieve crystallization. JP also conversed with an impartial researcher as a sounding board; the nature of the conversations was to explore the dynamic relationship between the researchers and the topic of research (Janesick, 2001). The aim of these conversations was to enable us to present a more thorough and authentic study (Janesick, 2001).

### Ethical Issues

In conducting the present research, two participants engaged in interviews over the telephone which may have resulted in a feeling of disconnection from the researcher as they could not be seen (Hanna, 2012). Although the participants did not make this notion explicit, the researcher acknowledges the potential limitations of this mode of communication. To honour the respect and dignity of all participants by giving them the chance to be heard, valued and speak freely knowing the researcher is paying respectful attention, the researcher endeavoured to build rapport in other ways (e.g., friendly conversation before the interview describing where the researcher was and her intention to listen even though she could not be seen).

## Results

**FIGURE 1:**
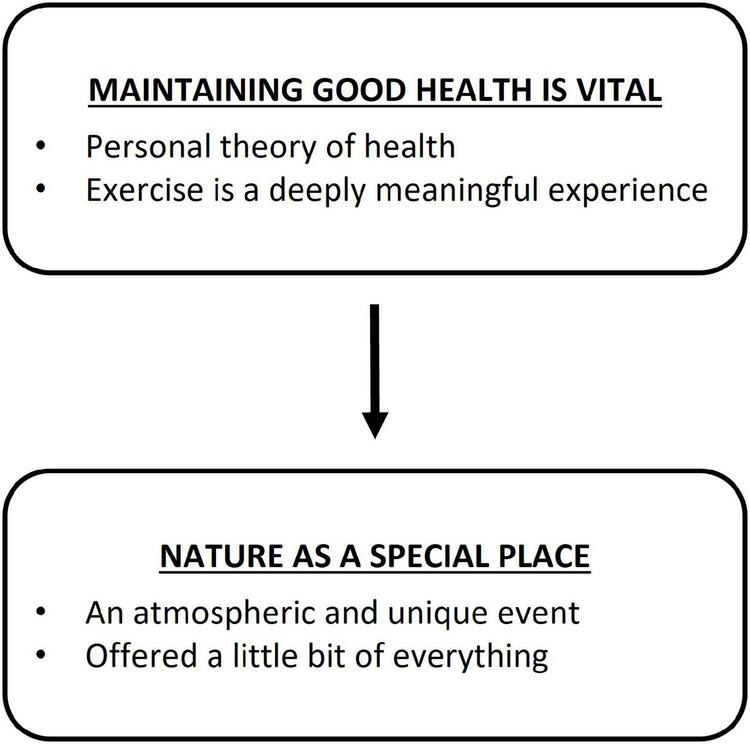
Thematic map of superordinate themes.

The aim of this investigation was to explore the lived experiences of people who participated in a nature-based running event. Through the methodological process previously outlined, two superordinate themes were developed: (1) maintaining good health is vital; and (2) nature as a special place. In broad terms, “maintaining good health is vital” encapsulated participants’ intrinsic drive to develop and maintain all aspects of their health including physical, emotional, and mental. Accordingly, this superordinate theme informed the discovery of the second superordinate theme. “Nature as a special place” encapsulated the participant’s perceptions of the event as containing a unique community and atmosphere that also offered them a little bit of everything (i.e., a wide variety of technical challenges, and environmental diversity). In this way, the natural environment describes participant’s perceptions of the special, novel and affording aspects of the Otway Ranges where the run took place. Maintaining good health is vital is identified as the overarching theme; this theme served as the catalyst to register for the nature-based event. This hierarchal relationship is demonstrated in Figure 1.

### Maintaining Good Health Is Vital

The experience of participating in the trail running event served as an example of the strong desire to maintain good health. In the context of this study, health is understood as a positive concept that incorporates physical capabilities, social, and personal resources that enable an individual to reach their full potential. Participants were unanimous in their view that health was something that only they could manage and possessed a strong desire to maintain, including undertaking novel events such as the Forrest trail run. Within this superordinate theme were two higher order themes: personal theory of health; benefits of exercise.

#### Personal Theory of Health

Exercising was a critical aspect of maintaining health, with all participants reflecting on the personal relevance. For some participants, partaking in exercise provided a sense of freedom and control that was a consequence of deliberately attempting to overcome ill health. Whether the ill health was physical or mental in orientation, participants utilised exercise as a form of self-initiated treatment. Hence the personal theory of health reflected the process of engaging in exercise. For example, Mike spoke candidly about achieving a sense of control and freedom following the decision to take responsibility of his physical health by starting to run:

I’m 65 years old now. When I was 57, my eldest brother died of a sudden heart attack and as a result of that, the suggestions from the doctor was that maybe people should go and have a bit of a check-up and find out why. At that stage I weighed about 110 kilos and I smoked anywhere between 20, 40 cigarettes a day…. And drank a hell of a lot of coffee with two sugars in it. So they said I needed to do something, I found that exercising in the park wasn’t fantastic. I tried lots of different things, some combative exercises and things like that. And finally I fell into running. And I just love it because running what it means to me is that for half an hour, 20 min, whatever, I’m on my own…No one can tell me what to do, where to go or anything at all. And I love it. (Mike)

This excerpt captures a dynamic relationship between Mike’s health concept (e.g., actively acknowledging health risks such as being overweight, smoking and sugar intake and how that might impact his lifespan), and Mike’s agency and responsibility regarding his health by using exercise as a means to combating these risk factors. The meaning Mike ascribes to the action of running reveals it as an enjoyable experience that enables him to exert control in his life. The freedom and control that running permits Mike, also allows him to improve his physical and emotional wellbeing whilst taking responsibility of his health.

For other participants, their personal theory of health was intrinsically tied with the concept of seeking challenges; by looking for opportunities to challenge themselves and their bodies, they would be able to better maintain health. Jane, for example, entered the event for the first time knowing that it was a challenging course. When recounting some of the more difficult aspects, Jane recalled how she drew inspiration from other runners:

I met a blind lady [on the run and] she can’t see what she’s doing, but she’s doing it. It was amazing. She made me think “Wow, if she could do it, I could finish this.” To me, the thing that stuck out the most [was that she] was doing it that was blind. It was just amazing to see she was doing it with a helper… That really spurred me on. I thought, “I’ve got nothing wrong with me at all.”

As a mature participant and novice at the event, the encounter with the blind runner enabled Jane to reassess her own abilities; whilst the run was challenging for Jane, she realised that it was also achievable. This chance encounter led to a new sense of perspective and motivation for Jane, which served as confirmation that challenging activities can be rewarding for her health.

Under this broader theme of personal health, it was also apparent that the decision to be physically active and responsible for one’s health was a personally initiated choice to better manage their mental health. The importance of exercise in facilitating these outcomes was summarised by Susan when describing her personal theory for exercise:

So my theory was if I went for a run, I could have a couple of bits of chocolate. So it was a bit about balance as well for me. Probably, if anything, my running and my exercise has increased a little bit because of things that happened at home in terms of social, or not social, personal life. So therefore running was an escape. And probably that greater correlation between the mindset and the feel good. You go for a run, you feel a little bit better. I think if I didn’t turn to running, I possibly could have ended up on antidepressants, so I haven’t gone down that path. I see a psychologist and everything like that regularly, and sort of running has now, and exercise, has sort of been that, not the happy place, but a place that makes you feel better. An escape, you know? It just, and it’s just a part of good routine as well. (Susan)

Here, Susan’s experience reveals her personal theory of health that guides her exercise behaviour. This theory includes the idea that actively engaging in running can justify an enjoyable behaviour that she may see as detrimental to her idea of health (e.g., eating some chocolate). For Susan, her personal life and mental health are important contributing factors that led her to run and remain a strong source of motivation. In this sense, Susan runs to simultaneously facilitate enjoyable experiences, and avoid unpleasant feelings, her push-pull relationship with running underscores her personal theory of health. The significance of running for Susan suggests that it holds personal meaning that intertwines her physical, emotional, and mental wellbeing, allowing her to both manage and take responsibility of her overall state of health.

#### Exercise Is a Deeply Meaningful Experience

As exercise was an important resource for improving health, most participants recalled deeply meaningful benefits associated with exercise. These benefits encompassed the outcomes associated with performing exercise, such as those evident in Mike’s experiences following his decision to commence running to improve his physical health. For other participants, such as Molly, running also resulted in benefits to mental health:

It’s like a meditation, running. It’s like a form of processing everything that’s going on in your brain… The feel good, the endorphins after exercising are great…And even the group that I run with as well, the trail running, great people. Just nice to get out and socialize. And I’ve been through some pretty challenging times the last few years. And I’ve actually mentioned to the guy that does the group training and I said to him, “If I hadn’t come… If I hadn’t had you and the class…” Yep, anyway…. Really, really good for my mental health. (Molly)

Molly’s description of emotional, mental, and physical benefits of running attests to the holistic benefits of maintaining exercise. Running allowed Molly to process her thoughts, feel the positive effects of endorphins, and socialise. Whilst not explicitly stated, Molly alludes to potential negative consequences of not participating in the group training. Molly’s experiences suggest that she needs to exercise to maintain good mental health. Together, these beneficial outcomes helped Molly to get through personal challenges in a meaningful way such that running has become a resource that she actively utilises to improve and maintain her mental health.

### Nature as a Special Place

This superordinate theme resembled a context that was understood as novel, different, and personally meaningful for all participants. Following the analysis of individual experiences, two higher order themes were constructed that represented the atmospheric and uniqueness of the event, and that the running trail itself offered so much variety to participants. Regarding the natural environment, the event was geographically located in the Otway ranges of Australia. Many participants described the environment as very different to other natural environments that they had witnessed both during other events and in their everyday lives. Common descriptions included statements such as “just so beautiful,” “so different,” “so pretty and green,” and “like nothing seen before.” Attributes such as the local wildlife, varying terrains, many different types of flora, and colours and smells stood out as novel and endearing features. One rich description of the personally meaningful value attributed to the natural environment can be seen here as Susan recounted what she found so beautiful about the lake:

It’s funny…it’s [the running trail environment] sort of got this…mystical feeling about it. It could be quite eerie at times when, I know when I’d been out there the week before, it was wet and cold and raining, and that’s why I say it was, had a bit of a mystical feel about it. It was very dark. And there’s these stumps that come out, dead trees that come out of the water. It’s a pretty cool old place. But then it could be quite a beautiful place when the sun comes out and the blue sky. So it just sort of took on these multiple different personas, I guess, it adapted, had different personalities essentially. (Susan)

Here, Susan describes the external environment with rich emotion. The lake adjacent to the running trail seemingly takes on familiar personalities commonly attributed to humans. For Susan, the lake may be interpreted as a reflection of various mood states. In this sense, the lake is more than a lake as an outside stimuli, it is a place that is ascribed a special personal meaning for which Susan connects her unique experience of human emotion.

#### An Atmospheric and Unique Event

It was unanimous that attending the event resulted in experiencing a strong sense of community and positive atmosphere. Here, atmosphere not only included the feeling participants’ experienced in the start area prior to commencing the event, but also the feeling of being immersed in the natural surrounds. The community feel was characterised by novel features of the event itself, such as fire pits to huddle around prior to the start of the event (the event was scheduled during the Australian winter), and the types of people who participated in the event (e.g., people in various costumes, a blind lady and her guide, and various types of trail runners). As an indication of how unique the event was, Rachel explained how the Run Forrest event differed from other events she had previously completed:

I guess, part of that having a much smaller event that does feel more intimate and you feel perhaps a little more connected with the rest of the people and I think just the fact that it is trail running and there were clearly people there who had done a lot of trail runs before and there was an atmosphere of community. People knew each other from running at that event. And it felt like I was part of that, so that was very different in that respect to the other events that I’ve done where you feel like you’re just one of many thousands of people and you kind of stick with your group a little bit more. I mean, we stuck with our group as well at that event, but it did feel like we were in more of a community. (Rachel)

As her first trail running event, Rachel noticed a feeling of connection and a sense of community; Rachel was able to identify with those around her and was provided with a sense of belonging that was absent at other events. Rachel embraced the opportunity to be a part of this positive atmosphere in a personally meaningful way, adding greater value to her first-time experience.

In the year of data collection, the event was also held over a long weekend, which for many participants allowed them the opportunity for time away. In describing what made her sign up for the event, Rachel elucidated how the event afforded her the opportunity for time away:

I think when Run Forrest came up I realized that… we had a friend’s place nearby that we could stay at, so I suggested a weekend away with these two girls that I run with often. And so it was a bit of an excuse to go away and have a weekend with friends but also to try trail running which we thought it would be a fun experience… I think part of the fun of it for us was the fact that it was a destination and so it demanded us to have a bit more timeout and we made a whole weekend around that event. Whereas if we had run the Run Melbourne event [festival of short and mid-distance running events located in the Melbourne central business district], for example, we would get together for that event and then maybe probably have breakfast together and then go our separate ways. So it’s a much shorter, social event for me. Then based on which ended up being two nights away, which is great. (Rachel)

This excerpt demonstrates Rachel’s idea of the event as being more than a timed race. For Rachel, the event is an opportunity for exercise, fun, social interaction, and a short holiday. The event in this sense is a destination that afforded time out and enabled Rachel to experience many different things that she considered personally meaningful. Other participants also reported similar experiences, with one participant describing her decision to travel 750 kilometres to attend the event. For Molly, the opportunity to exercise, spend time with her daughter, challenge herself, and be in nature were personally meaningful values that she felt driven to travel to experience. Many of the participants had also arranged accommodation to spend the long weekend in Otway and engage in the extra activities available in the town (i.e., a shorter running event the day before, and a soup festival following the Run Forrest event).

#### Offered a Little Bit of Everything

It was apparent that the actual trail run itself held significant meaning for all participants, who spoke of the diverse landscape and the challenges, distractions, and enjoyable experiences it provided. Sally’s recollection of the environmental diversity best exemplified this theme:

There was a bit of everything, and I think that’s what I really liked about it as well, and it sort of distracts you from the challenging elements of it. So at some points you had wider gravelly, a wider gravelly hill where you could have maybe half a dozen people across. Other times it was sort of a narrower, windy, muddy track where you might run single file, and I think what was really nice, even with that single file, is that… I slipped and some woman behind… not slipped properly but just slightly, and some woman behind me joked, in a nice way, keeping an eye out, and then someone else was a bit slow in front of me and she encouraged me to go round her. I don’t think I’ve ever experienced anything like that in road running. It’s different because obviously you’re not on a tiny little track like that. So, yeah, the terrain was very varied, and, like I said, I think that was good because it was quite distracting. So there seemed to be a bit of everything. It was nice to run over those little wooden footbridges, or something, with the big ferns. (Sally)

In Sally’s case, the constantly changing terrain was both personally engaging and challenging. More importantly, the natural environment afforded Sally a novel experience she had never previously encountered, despite being a seasoned runner. The exposure meant that Sally was continuously reassessing her surrounds and the novel situations which she found herself running through. This environment also provided many opportunities for interactions with others and a sense of enjoyment.

All participants recalled distinct aspects of the environment that resonated with them individually. Whilst many participants had previously competed in large scale events (e.g., marathons, cycling events), for all participants, this was their first time running in this event. All participants commented that they were drawn to this event in some way, and that the event was an achievement of great value. In describing a part of the race that stood out to her, Sally commented that:

I’ve never seen anyone stop and take a picture of anything mid race or run, apart from some park runs, but if you’re looking at a timed event, no, I don’t think I’ve seen that before, and people just seemed, yeah… It’s hard to describe but it was more of a relaxed vibe, friendlier vibe. (Sally)

As a regular runner, Sally experienced a degree of surprise when noticing other competitors stopping to take a photograph of a koala during the timed event. This experience epitomised the event vibe for Sally such that seeing others prioritise the experience of taking in novel stimuli rather than focussing on individual performance (e.g., achieving a good run time) added to a positive perception of the event. In this sense, the event afforded her an opportunity to vicariously appreciate nature, which in turn reinforced the concept of it being a friendly and relaxed event.

For some of the regular runners in this research, the track also included novel technical challenges that they had to navigate. For instance, Molly and Sally both described the new experience of having mud accumulating on the bottom of their shoes and the associated physical toll it had on their bodies (e.g., feeling heavy and shoes being slippery).

…I found that last couple of kilometres really difficult. My shoes were really heavy with mud, I think, and I don’t know how to …My legs just felt really heavy and my feet felt really heavy which I’ve never really experienced before. But then I was thinking, I’ve only got a couple of kilometres to go, it’s fine. So even though I was finding it physically a little bit hard, I knew I didn’t have that far to go. (Sally)

In this sense, the dynamic environment (e.g., wet dirt tracks, wooden bridges, and concrete paths) ensured that participants were faced with many different conditions that required constant adjusting, learning, and skill development which for many were enjoyable experiences. For Rachel, the natural environmental guided conversations between her and her friends during the event creating an enjoyable experience and a distraction from running. For others like Molly and Sally, the natural environment was a place that they described as the most beautiful and different place they had seen, making the event a more pleasurable place to take on the challenge. For Mike, it was the first time he perceived the environment as undiscriminating. This was a fascinating experience for Mike as he described the environment as demanding cooperation to navigate, resulting in experiences of comradery and learning that he had not witnessed before. It was also a place he felt inclined to share with the researcher by sending in photographs a day following his interview.

## Discussion

The aim of this research was to explore the lived experiences of people who participated for the first time in a nature-based trail running event using an interpretive phenomenological stance. Following data analysis, we put forward two superordinate themes that represented participant experiences: (1) maintaining good health is vital; and (2) nature as a special place. Actively engaging in exercise was an integral means by which participants were intrinsically motivated to both promote, and mediate, their overall health. It was also a primary factor leading participants to take part in the event. The event itself provided unique value adding opportunities that initiated and maintained engagement and enjoyment at the event. Furthermore, the natural environment was considered a diverse, novel, and special place, suggesting nature is perceived as a personally favourable and meaningful environment by these participants. Collectively, these findings suggest that experienced runners participating in a trail running event afforded the individuals a personally meaningful experience that was aligned with their motivation to exercise.

Results complement existing work in sports, exercise, and natural environment literature. Regarding the maintaining good health is vital superordinate theme, participants’ self-initiated engagement with exercise to control personal situations and improve overall health is consistent with the self-efficacy and self-mastery attributes of physical activity (see Netz et al., 2005). Moreover, participants’ motivating factors that contributed to entering Run Forrest are also consistent with participants’ motivation to visit green spaces, whereby park users in the United Kingdom were motivated to visit green spaces for physical pursuits (e.g., walking, exercise; Irvine et al., 2013). The positive aspects of nature mentioned by participants (e.g., green scenery, types of plants, and views found pleasing) is also consistent with research demonstrating a like and preference for the natural environment (Ulrich, 1983, 1999; Wilson, 1984; Kaplan and Kaplan, 1989). The personal benefits associated with exercise, as reported by current participants, are also congruent across diverse populations; individuals with severe mental illness experienced improved mood as a consequence of becoming more active (Firth et al., 2016), while runners can potentially increase their life expectancy by 3.2 years compared to non-runners (Lee et al., 2017).

Similarly, there were parallels between our findings and those reported in the emerging *parkrun* literature. For example, the need to maintain health is comparable to the desire to participate in *parkrun* to improve individual mental health (Dunne et al., 2021). Likewise, participants’ perceptions of an atmospheric community at the event appears analogous to the sense of community connectedness experienced at *parkrun* (Grunseit et al., 2017; Davis et al., 2021). Collectively, the alignment between the current findings and existing research regarding motivation towards maintaining good health in natural environments reinforces the virtue that these contexts are beneficial to our everyday functioning.

In addition to the consistencies mentioned above, as the first known qualitative investigation of a nature-based trail running event, this research also contributes some novel findings in two ways. First, the subjective and complex interactions between participants and their respective experiences at this event highlight the psychological, emotional, and physical components involved in nature-based exercise as informed by EDP. For example, the rich descriptions of the participants’ individual and unique characteristics (i.e., being older, first time in trail running environment, running alone) and the ways in which they navigated these factors to complete the event demonstrate active engagement with both internal (i.e., psychological) and external (i.e., the varying terrain and other competitors) components during the run. For many participants, this active engagement manifested as a challenging experience that resulted in a considerable sense of achievement and enjoyment. Second, descriptions of the unique environment add support to the EDP concept of affordances; a narrow running trail afforded some participants the opportunity to “crash through” whereas others slowed down to “enjoy the greenery.” Regardless of the individual affordance, the sense of camaraderie and friendship among fellow runners was noticeably different in this event compared to larger events. In this respect, the opportunity for social inclusion and group identification evident among our participants, was similar to freedom and reciprocity attributes among other community-based events such as music festivals (Ballantyne et al., 2014).

More broadly, these affordances presented as opportunities for achievement, social connection, skill development, and time away. Given that nature-based adventurous activities (e.g., skydiving) can foster wellbeing by fulfilling basic psychological needs (MacKenzie and Brymer, 2018), it is plausible that the affordances within nature-based mass participation sporting events also contribute towards individual wellbeing. In other words, our findings imply that nature-based running events can hold important opportunities to facilitate individual wellbeing for those with prior experience in performing exercise.

### Theoretical and Methodological Implications

Possibly the most unique contribution of this study comes from the methodological design; maintaining an interpretivist paradigm and employing IPA were instrumental in yielding the reported findings for two reasons. First, the interpretivist paradigm allowed for an open-minded investigation into nature-based exercise. In this sense, the atheoretical analyses facilitated us as researchers to provide novel information regarding participants’ subjective experiences and avoid overlaying a preconceived notion of nature-based exercise. That the findings exemplify the notion of affordances (i.e., how participants engaged with the natural environment), further strengthens the EDP approach to nature-based research. Second, employing IPA allowed the researchers to directly explore the complex lived experiences of participants by gathering deep and contextual information surrounding the human-nature relationship and the values attributed to the event and environment. As such, this specific methodology has facilitated a thorough investigation which has allowed for the elicitation of the unique findings to support EDP.

The alignment between our findings and those reported in the existing literature also sit well with the current generalizability debate among qualitative researchers (e.g., Smith, 2018). Specifically, the congruence between the current findings on the necessity to maintain good health and those from previous investigations (e.g., Kwasnicka et al., 2016) suggest a degree of naturalistic generalisability; our findings may resonate with the implicit experiences of green exercise researchers and consumers (Barton and Pretty, 2010). Similarly, it would be prudent for future research to investigate the transferability of the current findings (Lewis, 2014). Specifically, participants in the present study had prior experience in performing exercise hence their stories are reflective of individuals with self-determined motives to undertake a personally unique running event. However, these experiences may naturally differ to those who may be new to running and the trail running event altogether. Given that the construction of knowledge is subjective and can represent multiple realities (Smith, 2018), and that the proliferation of nature-based recreation activities abounds (Mackintosh et al., 2018), it would be wise to continue the investigation of such trail running events within nature with a particular focus on participants’ motivation, ableism, and the human-environment relationship. Whilst beyond the scope of the current study, future investigations should include an exploration of the barriers and facilitators for individuals and or groups who may be less likely to undertake nature-based exercise, particularly such trail running events. Findings from research that focuses on these individuals and groups may help us to better understand how we can improve accessibility and engagement with nature-based exercise more broadly across society. Furthermore, there is mixed research regarding the impact of urban mass participation sporting events on sustained health behaviour change; such events may not facilitate a shift in long-term motivation to exercise (Coleman and Sebire, 2016). If future research was to consolidate the current findings by examining long-term engagement in nature-based events, then that evidence may reveal strategies to better support individuals to change exercise-related behaviour. Such research would move the green exercise field beyond the romanticised relationship and into one that showcases a deeper understanding of when, how, where, and why nature-based exercise can benefits individuals and communities.

## Conclusion

In summary, this research aimed to explore the lived experiences of people who participated in a nature-based mass participation running event. An in-depth analysis of six individuals’ experiences aided in the construction of two superordinate themes: maintaining good health is vital, and nature as a special place. These themes resembled participants’ personal theory of maintaining health behaviour (i.e., physical, emotional, mental), and their connection with nature (i.e., a special and novel environment). Collectively, these findings suggest that engaging in a nature-based event is beneficial, to the point that completing a nature-based running event afforded participants novel encounters that may have contributed towards their individual wellbeing. Future research could continue this line of inquiry by examining longer-term and repetitive engagement with the natural environment, such as frequently participating in similar events. Findings from these investigations would contribute towards a better understanding of how mass-participation sporting events in nature may enable long-term health behaviour change.

## Data Availability Statement

The raw data supporting the conclusions of this article will be made available by the authors, without undue reservation.

## Ethics Statement

The studies involving human participants were reviewed and approved by Navitas Professional Institute Human Research Ethics Committee. The patients/participants provided their written informed consent to participate in this study.

## Author Contributions

TC conceived and designed the study and wrote various sections of the manuscript. JP recruited the participants, conducted all the interviews, and wrote the first draft of the manuscript. Both authors performed the data analysis, read, and approved the submitted version.

## Conflict of Interest

The authors declare that the research was conducted in the absence of any commercial or financial relationships that could be construed as a potential conflict of interest.

## Publisher’s Note

All claims expressed in this article are solely those of the authors and do not necessarily represent those of their affiliated organizations, or those of the publisher, the editors and the reviewers. Any product that may be evaluated in this article, or claim that may be made by its manufacturer, is not guaranteed or endorsed by the publisher.

## References

[B1] Akers A. Barton J. Cossey R. Gainsford P. Griffin M. Micklewright D. (2012). Visual color perception in green exercise: positive effects on mood and perceived exertion. *Environ. Sci. Technol.* 46 8661–8666. 10.1021/es301685g 22857379

[B2] Araujo D. Brymer E. Brito H. Withagen R. Davids K. (2019). The empowering variability of affordances of nature: why do exercisers feel better after performing the same exercise in natural environment than in indoor environments? *Psychol. Sport Exerc.* 42 138–145. 10.1016/j.psychsport.2018.12.020

[B3] Badbury-Jones C. (2007). Enhancing rigor in qualitative health research: exploring subjectivity through peshkin’s I’s. *J. Adv. Nurs.* 59 290–298. 10.1111/j.1365-2648.2007.04306.x 17590210

[B4] Ballantyne J. Ballantyne R. Packer J. (2014). Designing and managing music festival experiences to enhance attendee’s psychological and social benefits. *Sage J.* 18 65–83. 10.1177/1029864913511845

[B5] Barton J. Pretty J. (2010). What is the best dose of nature and green exercise for improving mental health? A multi-study analysis. *Environ. Sci. Technol.* 44 3947–3955. 10.1021/es903183r 20337470

[B6] Bauman A. Murphy N. Lane A. (2009). The role of community programmes and mass events in promoting physical activity to patients. *Br. J. Sports Med.* 43 44–46. 10.1136/bjsm.2008.054189 18971245

[B7] Berger R. (2015). Now i see it, now i don’t: researcher’s position and reflexivity in qualitative research. *Qual. Res.* 15 219–234. 10.1177/1468794112468475

[B8] Berman M. G. Jonides J. Kaplan S. (2008). The cognitive benefits of interacting with nature. *Psychol. Sci.* 19 1207–1212. 10.1111/j.1467-9280.2008.02225.x 19121124

[B9] Berman M. G. Kross E. Krpan K. M. Askren M. K. Burson A. Deldin P. J. (2012). Interacting with nature improves cognition and affect for individuals with depression. *J. Affect. Disord.* 140 300–305. 10.1016/j.jad.2012.03.012 22464936 PMC3393816

[B10] Boland R. J. (1985). “Phenomenology: a preferred approach to research on information systems,” in *Research Methods in Information Systems*, eds Mumford E. Hirschheim R. A. Fitzgerald G. Wood-Harper A. T. (Amsterdam: North-Holland), 193–201.

[B11] Bratman G. N. Anderson C. B. Berman M. G. Cochran B. de Vries S. Flanders J. (2019). Nature and mental health: an ecosystem service perspective. *Sci. Adv.* 5 1–14. 10.1126/sciadv.aax0903 31355340 PMC6656547

[B12] Braun V. Clarke V. (2013). *Successful Qualitative Research: A Practical Guide For Beginners.* London: Sage.

[B13] Buning R. J. Walker M. (2016). Differentiating mass participant sport event consumers: traditional versus non-traditional events. *Sport Mark. Q.* 25 47–58.

[B14] Cacioppo J. T. Semin G. R. Bernston G. G. (2004). Realism, instrumentalism, and scientific symbiosis: psychological theory as a search for truth and the discovery of solutions. *Am. Psychol.* 59 214–233. 10.1037/0003-066X.59.4.214 15149262

[B15] Chang Y. Pan C. Chen F. Tsai C. Huang C. (2012). Effect of resistance-exercise training on cognitive function in healthy older adults: a review. *J. Aging Phys. Act.* 20 497–517. 10.1123/japa.20.4.497 22186664

[B16] Cohen L. Manion L. Morrison K. (2007). *Research Methods In Education*, 6th Edn. London: Routledge.

[B17] Coleman S. J. Sebire S. J. (2016). Do people’s goals for mass participation sporting events matter? A self-determination theory perspective. *J. Public Health* 39 202–208. 10.1093/pubmed/fdw090 27679656

[B18] Coon J. T. Boddy K. Stein K. Whear R. Barton J. Depledge M. H. (2011). Does participating in physical activity in outdoor natural environments have a greater effect on physical and mental wellbeing than physical activity indoors? A systematic review. *Environ. Sci. Technol.* 45 1761–1772. 10.1021/es102947t 21291246

[B19] Cowan D. Taylor I. M. (2016). ‘I’m proud of what I achieved; I’m also ashamed of whatI done’: A soccer coach’s tale of sport, status, and criminal behaviour. *Qual. Res. Sport Exerc. Health* 8 505–518. 10.1080/2159676x.2016.1206608

[B20] Creswell J. W. (1998). *Qualitative Inquiry And Research Design: Choosing Among The Five Traditions.* Thousand Oaks, CA: Sage.

[B21] Creswell J. W. (2009). *Research Design: Qualitative And Mixed Methods Approaches.* London: SAGE.

[B22] Crotty M. (1998). *The Foundations Of Social Research.* London: Sage.

[B23] Davis A. J. MacCarron P. Cohen E. (2021). Social reward and support effects on exercise experiences and performance: evidence from parkrun. *PLoS One* 16:e0256546. 10.1371/journal.pone.0256546 34525097 PMC8443045

[B24] Ding D. Lawson K. D. Kolbe-Alexander T. L. Finklestein E. A. Katzmarzyk P. T. van Mechelen W. (2016). The economic burden of physical inactivity: a global analysis of major non-communicable diseases. *Lancet* 388 1312–1324. 10.1016/S0140-6736(16)30383-X 27475266

[B25] Dunne A. Haake S. Quirk H. Bullas A. (2021). Motivation to improve mental wellbeing *via* community physical activity initiatives and the associated impacts-a cross-sectional survey of UK parkrun participants. *Int. J. Environ. Res. Public Health* 18 1–13. 10.3390/ijerph182413072 34948683 PMC8702167

[B26] Eatough V. Smith J. A. (2008). “Interpretative phenomenological analysis,” in *The Sage Handbook Of Qualitative Research In Psychology*, eds Willig C. Stainton-Rogers W. (London: Sage), 179–194.

[B27] Edge J. Richards K. (1998). May I see your warrant please? Justifying outcomes in qualitative research. *Appl. Linguist.* 19 334–356. 10.1093/applin/19.3.334

[B28] Firth J. Rosenbaum S. Stubbs B. Gorczynski P. Yung A. R. Vancmpfort D. (2016). Motivating factors and barriers towards exercise in severe mental illness: a systematic review and meta-analysis. *Psychol. Med.* 46 2869–2881. 10.1017/S0033291716001732 27502153 PMC5080671

[B29] Fraser M. Munoz S.-A. MacRury S. (2019). Does the mode of exercise influence the benefits obtained by green exercise? *Int. J. Environ. Res. Public Health* 16:3004. 10.3390/ijerph16163004 31434352 PMC6720300

[B30] Frumkin H. Bratman G. N. Breslow S. J. Cochran B. Kahn P. H. Lawler J. J. (2016). Nature contact and human health: a research agenda. *Environ. Health Perspect.* 127 1–18. 10.1289/EHP1663 28796634 PMC5744722

[B31] Gadamer H. G. (1976). *Philosophical Hermeneutics (D. E. Linge, Trans.).* Berkeley: University of California Press.

[B32] Gibson J. J. (1979). *The Ecological Approach to Visual Perception.* Boston, MA: Houghton Mifflin.

[B33] Grinde B. Patil G. G. (2009). Biophilia: does visual contact with nature impact on health and well-being? *Int. J. Environ. Res. Public Health* 6 2332–2343. 10.3390/ijerph6092332 19826546 PMC2760412

[B34] Grix J. (2004). *The Foundations Of Research.* London: Palgrave Macmillan.

[B35] Grunseit A. Richards J. Merom D. (2017). Running on a high: parkrun and personal well-being. *BMC Public Health* 18:59. 10.1186/s12889-017-4620-1 28743304 PMC5526231

[B36] Guba E. G. Lincoln Y. S. (1994). “Competing paradigms in qualitative research,” in *Handbook of Qualitative Research*, eds Denzin N. K. Lincoln Y. S. (London: Sage), 105–117.

[B37] Hanna P. (2012). Using internet technologies (such as Skype) as a research medium: a research note. *Qual. Res.* 12 239–242. 10.1177/1468794111426607

[B38] Immonen T. Brymer E. Davids K. Liukkonen J. Jaakkola T. (2018). An ecological conceptualization of extreme sports. *Front. Psychol.* 9:1274. 10.3389/fpsyg.2018.01274 30087641 PMC6066723

[B39] Irvine K. N. Warber S. L. Devine-Wright P. Gatson K. J. (2013). Understanding urban green spaces as a health resource: a qualitative comparison of visit motivation and derived effects among park users in Sheffield, UK. *Int. J. Environ. Res. Public Health* 10 417–442. 10.3390/ijerph10010417 23340602 PMC3564151

[B40] Janesick V. J. (2001). Intuition and creativity: a pas de deux for qualitative researchers. *Sage J.* 7 531–540. 10.1177/107780040100700501

[B41] Josefsson T. Lindwall M. Archer T. (2013). Physical exercise intervention in depressive disorders: meta-analysis and systematic review. *Scand. J. Med. Sci. Sports* 24 259–272. 10.1111/sms.12050 23362828

[B42] Joye Y. de Block A. (2011). ‘Nature and I are two’: a critical examination of the biophilia hypothesis. *Environ. Values* 20 189–215. 10.3197/0963271111X12997574391724

[B43] Kaplan R. Kaplan S. (1989). *The Experience of Nature: A Psychological Perspective.* New York, NY: Cambridge University Press.

[B44] Kjellgren A. Buhrkall H. (2010). A comparison of the restorative effect of a natural environment with that of a simulated natural environment. *J. Environ. Psychol.* 30 464–472. 10.1016/j.jenvp.2010.01.011

[B45] Kwasnicka D. Dombrowski S. U. White M. Sniehotta F. (2016). Theoretical explanations for maintenance of behaviour change: a systematic review of behaviour theories. *Health Psychol. Rev.* 10 277–296. 10.1080/17437199.2016.1151372 26854092 PMC4975085

[B46] Lahart I. Darcy P. Gidlow C. Calogiuri G. (2019). The effects of green exercise on physical and mental wellbeing: a systematic review. *Int. J. Environ. Res. Public Health* 16:1352. 10.3390/ijerph16081352 30991724 PMC6518264

[B47] Lawton E. Brymer E. Clough P. Denovan A. (2017). The relationship between the physical activity environment, nature relatedness, anxiety, and the psychological well-being benefits of regular exercisers. *Front. Psychol.* 8:1058. 10.3389/fpsyg.2017.01.58 PMC5483473 28694788

[B48] Lee D. Brellenthin A. G. Thompson P. D. Sui X. Lee I.-M. Lavie C. J. (2017). Running as a key lifestyle medicine for longevity. *Prog. Cardiovasc. Dis.* 10.1016/j.pcad.2017.03.005 28365296

[B49] Lee K. E. Williams K. J. H. Sargent L. D. Williams N. S. G. Johnson K. A. (2015). 40-second green roof views sustain attention: the role of micro-breaks in attention restoration. *J. Environ. Psychol.* 42 182–189. 10.1016/j.jenvp.2015.04.003

[B50] Lester S. (1999). *An Ýntroduction to Phenomenology Research.* Taunton: Stan Lester Developments.

[B51] Lewis J. (2014). “Generalizing from qualitative research,” in *Qualitative Research Practice*, 2nd Edn, eds Ritchie J. Lewis J. McNaughton Nicholls C. Ormston R. (London: Sage), 347–366.

[B52] MacKenzie S. H. Brymer E. (2018). Conceptualising adventurous nature sport: a positive psychological perspective. *Ann. Leisure Res.* 23 79–91. 10.1080/11745398.2018.1483733

[B53] Mackintosh C. Griggs G. Tate R. (2018). Understanding the growth in outdoor recreation participation: an opportunity for sport development in the United Kingdom. *Manag. Sport Leisure* 23 315–335. 10.1080/23750472.2019.1595093

[B54] Martyn P. Brymer E. (2014). The relationship between nature relatedness and anxiety. *J. Health Psychol.* 21 1436–1445. 10.1177/1359105314555169 25370570

[B55] Mustafa R. F. (2011). The POEMs of educational research: a beginner’s concise guide. *Int. Educ. Stud.* 4 23–30.

[B56] Neidemeier M. Einwanger J. Hartl A. Kopp M. (2017). Affective responses in mountain hiking-A randomized crossover trial focusing on differences between indoor and outdoor activity. *PLoS One* 16:e0177719. 10.1371/journal.pone.0177719 28520774 PMC5433751

[B57] Netz Y. Wu M. J. Becker B. J. Tenenbaum G. (2005). Physical activity and psychological wellbeing on advanced age: a meta-analysis of intervention studies. *Psychol. Aging* 20 272–284. 10.1037/0882-7974.20.2.272 16029091

[B58] Olafsdottir G. Cloke P. Vogele C. (2017). Place, green exercise and stress: an exploration of lived experience and restorative effects. *Health Place* 46 358–365. 10.1016/j.healthplace.2017.02.006 28270319

[B59] Olive R. Wheaton B. (2021). Understanding blue space: sport, bodies, wellbeing, and the sea. *J. Sport Soc. Issues* 45 3–19. 10.1177/0193723520950549

[B60] Patton M. Q. (2002). *Qualitative Evaluation And Research Methods*, 3rd Edn. Newbury Park, CA: Sage.

[B61] Pretty J. Peacock J. Hine R. Sellens M. South N. Griffin M. (2007). Green exercise in the UK countryside: effects on health and psychological well-being, and implications for policy and planning. *J. Environ. Plann. Manag.* 50 211–231.

[B62] Pretty J. Peacock J. Sellens M. Griffin M. (2005). The mental and physical health outcomes of green exercise. *Int. J. Environ. Health Res.* 15 319–337. 10.1080/09603120500155963 16416750

[B63] Quirk H. Bullas A. Haake S. Goyder E. Graney M. Wellington C. (2021). Exploring the benefits of participation in community-based running and walking events: a cross-sectional survey of parkrun participants. *BMC Public Health* 21:1978. 10.1186/s12889-021-11986-0 34727918 PMC8561845

[B64] Reiner M. Niermann C. Jekauc D. Woll A. (2013). Long-term health benefits of physical activity – a systematic review of longitudinal studies. *BMC Public Health* 13:813. 10.1186/1471-2458-13-813 24010994 PMC3847225

[B65] Richardson L. (2000). New writing practices in qualitative research. *Sociol. Sport J.* 17 5–20.

[B66] Roe J. Aspinall P. (2011). The restorative benefits of walking in urban and rural settings in adults with good and poor mental health. *Health Place* 17 103–113. 10.1016/j.healthplace.2010.09.003 21094074

[B67] Rogerson M. Gladwell V. F. Gallagher D. J. Barton J. L. (2016). Influences of green outdoors versus indoors environmental settings on psychological and social outcomes of controlled exercise. *Int. J. Environ. Res. Public Health* 13:363. 10.3390/ijerph13040363 27023580 PMC4847025

[B68] Sandardos S. S. Chambers T. P. (2019). “It’s not about sport, it’s about you”: an interpretative phenomenological analysis of mentoring elite athletes. *Psychol. Sport Exerc.* 43 144–154. 10.1016/j.psychsport.2019.02.003

[B69] Schweitzer R. D. Glab H. Brymer E. (2018). The human-nature relationship: a phenomenological-psychoanalytic perspective. *Front. Psychol.* 9:969. 10.3389/fpsyg.2018.00969 29962988 PMC6010571

[B70] Scotland J. (2012). Exploring the philosophical underpinnings of research: relating ontology and epistemology to the methodology and methods of the scientific, interpretive, and critical research paradigms. *English Lang. Teach.* 5 9–16. 10.5539/elt.v5n9p9

[B71] Shanahan D. F. Bush R. Gaston K. J. Lin B. B. Dean J. Barber E. (2016). Health benefits from nature experiences depend on dose. *Sci. Rep.* 6 1–10. 10.1038/srep28551 27334040 PMC4917833

[B72] Sharman M. J. Nash M. Cleland V. (2019). Health and broader community benefit of parkrun-An exploratory qualitative study. *Health Promotion J. Australia* 30 163–171. 10.1002/hpja.182 29939453

[B73] Smith B. McGannon K. R. (2018). Developing rigor in qualitative research: problems and opportunities within sport and exercise psychology. *Int. Rev. Sport Exerc. Psychol.* 11 101–121. 10.1080/1750984X.2017.1317357

[B74] Smith J. A. (2017). Interpretative phenomenological analysis: getting at lived experience. *J. Positive Psychol.* 12 303–304. 10.1080/17439760.2016.1262622

[B75] Smith B. (2018). Generalizability in qualitative research: misunderstandings, opportunities and recommendations for the sport and exercise sciences. *Qual. Res. Sport Exerc. Health* 10, 137–149. 10.1080/2159676X.2017.1393221

[B76] Smith J. A. (2019). Participants and researchers searching for meaning: conceptual developments for interpretative phenomenological analysis. *Qual. Res. Psychol.* 16 166–181. 10.1080/14780887.2018.1540648

[B77] Smith J. A. Osborn M. (2008). “Interpretative phenomenological analysis,” in *Qualitative Psychology: A Practical Guide To Research Methods*, 2nd Edn, ed. Smith J. A. (London: Sage), 53–80.

[B78] Smith J. A. Flowers P. Larkin M. (2009). *Interpretative Phenomenological Analysis: Theory, Method And Research.* London: Sage.

[B79] Southern Exposure (2020). *The Otway Ranges Run Forrest.* St Pembroke Pines, FL: Southern Exposure.

[B80] Sparkes A. C. Smith B. (2009). Judging the quality of qualitative inquiry: criteriology and relativism in action. *Psychol. Sport and Exerc.* 10 491–497. 10.1016/j.psychsport.2009.02.006

[B81] Sparkes A. C. Smith B. (2014). *Qualitative Research Methods in Sport, Exercise, and Health: From Process to Product.* Abingdon: Routledge.

[B82] Stansen C. Chambers T. P. (2017). An interpretative phenomenological analysis of the player development manager role in Australian professional sports. *Qual. Res. Sport* 11 1–16. 10.1080/2159676X.2017.1397540

[B83] Stanton R. Reaburn P. (2013). Exercise and the treatment of depression: a review of the exercise program variables. *J. Sci. Med. Sport* 17 177–182. 10.1016/j.jsams.2013.03.010 23602562

[B84] Stevinson C. Wiltshire G. Hickson M. (2015). Facilitating participation in health-enhancing physical activity: a qualitative study of parkrun. *Int. J. Behav. Med.* 22 170–177. 10.1007/s12529-014-9431-5 25096794

[B85] Stewart H. Gapp R. Harwood I. (2017). Exploring the alchemy of qualitative management research: seeking trustworthiness, credibility and rigor through crystallization. *Qual. Rep.* 22 1–19.

[B86] Sullivan A. B. Scheman J. Venesy D. Davin S. (2012). The role of exercise and types of exercise in the rehabilitation of chronic pain: specific or nonspecific benefits. *Curr. Pain Headache Rep.* 16 153–161. 10.1007/s11916-012-0245-3 22258395

[B87] Thomas D. R. Hodges I. D. (2010). *Designing And Managing Your Research Project: Core Knowledge For Social And Health Researchers.* London: Sage.

[B88] Ulrich R. (1983). “Aesthetic and affective response to natural environment,” in *Human Behaviour and Environment*, Vol. 6 eds Altman I. Wohlwill J. (New York, NY: Plentum), 85–125. 10.1007/978-1-4613-3539-9_4

[B89] Ulrich R. S. (1999). “Effects of gardens on health outcomes: theory and research,” in *Healing Gardens. Therapeutic Benefits and Design Recommendations*, eds Cooper M. C. Barnes M. (New York, NY: John Wiley & Sons).

[B90] van den Berg A. E. Hartig T. Staats H. (2007). Preference for nature in urbanized societies: Stress, restoration, and the pursuit of sustainability. *J. Soc. Issues* 63 79–96. 10.1111/j.1540-4560.2007.00497.x

[B91] Visit Victoria (2020). *Forrest.* Available online at: https://www.visitmelbourne.com/Regions/Great-Ocean-Road/Destinations/Forrest (accessed March 9, 2020).

[B92] Warburton D. E. Nicol C. W. Bredin S. S. (2006). Health benefits of physical activity: the evidence. *Can. Med. Assoc. J.* 174 801–809. 10.1503/cmaj.051351 16534088 PMC1402378

[B93] WHO (2014). *Global Status Report On Noncommunicable Diseases.* Geneva: World Health Organization.

[B94] Wilson E. (1984). *Biophilia.* Harvard: Harvard University Press, 10.1145/1179849.1179879

[B95] Yeh H. Stone J. A. Churchill S. M. Brymer E. Davids K. (2017). Environmental benefits of different environments designed for treadmill running. *Environ. Res. Public Health* 14:752. 10.3390/ijerph14070752 28696384 PMC5551190

[B96] Yeh H. Stone J. A. Churchill S. M. Wheat J. S. Brymer E. Davids K. (2016). Physical, psychological and emotional benefits of green physical activity: an ecological dynamics perspective. *Sports Med.* 46, 947–953. 10.1007/s40279-015-0374-z 26330207

