# Supplementary material for: “Like Nothing I’ve Seen Before”: A Qualitative Inquiry Into the Lived Experience of Competing in a Trail Running Event
Source: Front Psychol. 2022 Mar 14;13:817685. doi: 10.3389/fpsyg.2022.817685 (PMC8963904; doi:10.3389/fpsyg.2022.817685)
Supplement: Supplementary file 1 [file Table_1.DOCX]

Trail running experience manuscript - Appendix A

Interview Questions

**Rapport building questions**

Can you tell me about your interest in running and exercise?

What other types of exercise do you like to do?

What types of places do you like to exercise in?

How do you feel exercise may impact mental health?

**Pre-run questions**

What led you to register for the Otway Forest Run?

Have you completed other nature-based events? If so, which ones and what were they like?

What type of training have you done?

What are some of the things you were most and least looking forward to?

What goals did you have going into this event?

**Post-run questions**

Can you walk me through your Otway experience?

What parts really stood out for you?

What was on your mind during the run?

How did you feel right before the run?

How did you feel after the run?

Do you think you met the goals that you had before the run?

Can you tell me about any difficult parts of the run?

How was the Otway run different from other events?

Now that the event is over, what’s changed?
